# Assessing Autophagy in Mouse Models and Patients with Systemic Autoimmune Diseases

**DOI:** 10.3390/cells6030016

**Published:** 2017-06-28

**Authors:** Fengjuan Wang, Baihui Li, Nicolas Schall, Maud Wilhelm, Sylviane Muller

**Affiliations:** 1 Centre National de la Recherche Scientifique (CNRS), Immunopathology and Therapeutic Chemistry/Laboratory of Excellence Medalis, Institut de Biologie Moléculaire et Cellulaire, Strasbourg 67000, France; Fengjuan.Wang@unistra.fr (F.W.); b.li@ibmc-cnrs.unistra.fr (B.L.); Nicolas.Schall@unistra.fr (N.S.); maud.wilhelm@hotmail.fr (M.W.); 2 University of Strasbourg Institute for Advanced Study (USIAS), Strasbourg 67000, France

**Keywords:** macroautophagy, chaperone-mediated autophagy, systemic lupus erythematous, Sjögren’s syndrome, autoimmunity, MRL/lpr mice, autophagy markers, salivary glands

## Abstract

Autophagy is a tightly regulated mechanism that allows cells to renew themselves through the lysosomal degradation of proteins, which are misfolded or produced in excess, and of damaged organelles. In the context of immunity, recent research has specially attempted to clarify its roles in infection, inflammation and autoimmunity. Autophagy has emerged as a spotlight in several molecular pathways and trafficking events that participate to innate and adaptive immunity. Deregulation of autophagy has been associated to several autoimmune diseases, in particular to systemic lupus erythematosus. Nowadays, however, experimental data on the implication of autophagy in animal models of autoimmunity or patients remain limited. In our investigations, we use Murphy Roths Large (MRL)/lymphoproliferation (lpr) lupus-prone mice as a mouse model for lupus and secondary Sjögren’s syndrome, and, herein, we describe methods applied routinely to analyze different autophagic pathways in different lymphoid organs and tissues (spleen, lymph nodes, salivary glands). We also depict some techniques used to analyze autophagy in lupus patient’s blood samples. These methods can be adapted to the analysis of autophagy in other mouse models of autoinflammatory diseases. The understanding of autophagy implication in autoimmune diseases could prove to be very useful for developing novel immunomodulatory strategies. Our attention should be focused on the fact that autophagy processes are interconnected and that distinct pathways can be independently hyper-activated or downregulated in distinct organs and tissues of the same individual.

## 1. Autophagy in Immunity and Autoimmune Diseases

**Figure 1:**
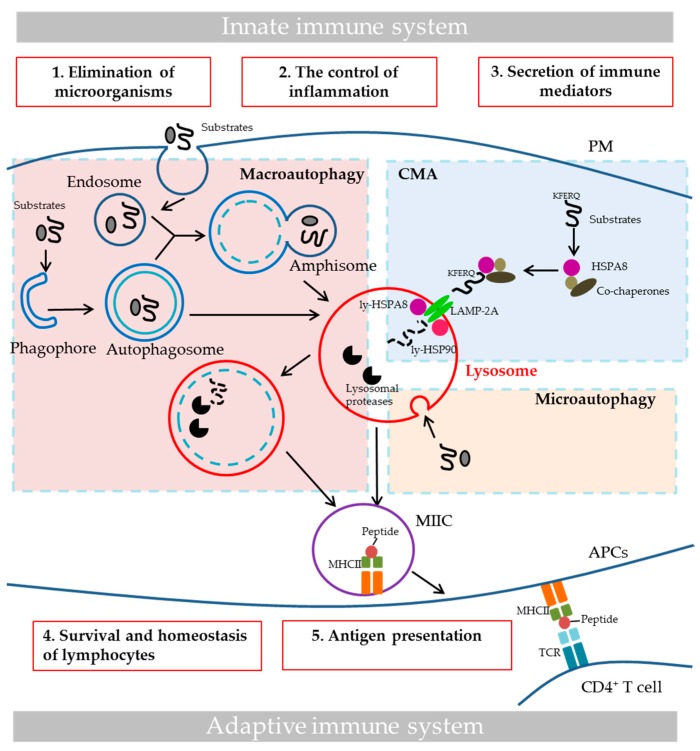
The three major autophagy pathways and the role of autophagy in immunity. The three principal bulk autophagy pathways are illustrated here: macroautophagy in the pink area of the figure, CMA in the blue area and microautophagy in the yellow area. The roles of autophagy in innate and adaptive immunity are highlighted in the red boxes. (**1**) Autophagy can directly eliminate invading microorganisms through xenophagy, MAP1LC3/LC3-associated phagocytosis (LAP), sequestosome-like receptors recruitement and still other scenarios [6]. (**2**) Autophagy controls inflammation, notably by affecting TLR signaling and suppressing inflammasome activation [19]. (**3**) Autophagy also controls inflammation through regulatory interactions with innate immune signaling pathways, via the removal of endogenous inflammasome agonists and through effects on the secretion of immune mediators, such as cathepsin K, lysozyme, IL-6, IL-8, damage-associated molecular patterns, etc. (**4**) Autophagy plays important role in T cell repertoire selection, maturation activation and polarization. Moreover, it is essential for the survival and function of B1 cells and plasma cells [20]. (**5**) As is illustrated here, autophagy participates in MHCII antigen presentation, and it could impact MHCI presentation as well [21]. APCs, antigen-presenting cells; CMA, chaperone-mediated autophagy; LAMP-2A, lysosomal-associated membrane protein type 2A; MAP1LC3/LC3, microtubule associated protein 1 light chain 3; ly-HSPA8 and ly-HSP90, lysosomal luminal HSPA8 and HSP90; MIIC, late endosomal MHC class II compartment; MHCI and II, major histocompatibility complex class I and II; IL-6 and -8, interleukin-6 and -8; PM, plasma membrane; TCR, T-cell receptor; TLR, toll-like receptor.

Autophagy is a cellular process, which removes unwanted cytoplasmic content, such as long-lived proteins, damaged organelles or invading microorganisms, via lysosomal degradation. Key features of this cytoprotecting process lead to cellular generation of energy and recycling of metabolic precursors. Three major pathways characterize bulk autophagy: macroautophagy (MaA), chaperone-mediated autophagy (CMA) and microautophagy (Figure 1). Other forms of autophagy exist that are more specific, e.g., mitophagy, which involves the selective degradation of mitochondria, lipophagy (degradation of lipids), and xenophagy, a type of selective autophagy that is used for eliminating invading pathogens. In MaA, a double membrane structure is formed to capture parts of cytosolic content to form the autophagosome, which further fuses with lysosomes to form the autolysosome in which the sequestered content is degraded [1] (see [2] for the nomenclature of autophagic vesicles). In CMA, specific substrates that contain the KFERQ-like motif are recognized by heat shock 70 kDa protein 8 (HSPA8/HSC70) chaperone protein, which targets the substrates to the surface of lysosomes and facilitates the binding of substrates to lysosome-associated membrane protein type 2A (LAMP-2A). The binding of substrates to LAMP-2A leads to the multimerization of the latter, and the translocation of substrates into lysosomal lumen, followed by their degradation by lysosomal proteases [3]. In microautophagy, substrates are trapped in vesicles formed by the invagination of lysosomal membrane, which are later pinched off from lysosomal membrane and degraded by lysosomal proteases [4].

Autophagy plays a broad spectrum of physiological roles, and acts decisively and in a coordinated, interconnected manner in various cellular processes, including immune processes. The role of autophagy in innate and adaptive immunity includes direct elimination of microorganisms, control of inflammation, secretion of immune mediators, control of homeostasis of immune cells and antigen presentation, which have been detailed in several excellent reviews [5,6,7,8,9,10,11,12] and summarized in Figure 1. The role of autophagy in major histocompatibility complex class II (MHCII) antigen presentation is illustrated in Figure 1. Using “loss- and gain- of-function” mutation-based experiments, both MaA and CMA have been demonstrated to play a role in MHCII antigen presentation. Deletion of proteins implicated in MaA, such as phosphoinositide 3-kinase (PI3K), autophagy-related 12 (Atg 12), autophagy-related 5 (Atg 5), compromises antigen presentation through MHCII and CD4^+^ T cell responses, while targeting antigens to autophagosomes through coupling microtubule associated protein 1 light chain 3B (MAP1LC3B; a MaA protein) to antigens dramatically increases their MHCII presentation [11,12,13,14,15]. Furthermore, pathogens, which inhibit MaA, are capable to escape from MHCII presentation [16,17]. Overexpression of LAMP-2A (CMA protein) has been found to be in favor of MHCII presentation of autoantigens [18]. Beside its role in MHCII antigen presentation, MaA has been reported to contribute to major histocompatibility complex class I (MHCI) presentation as well [11,12].

Autoimmune diseases represent a group of more than 100 illnesses in which the immune system shows breakdown of tolerance and consequently targets self-tissues and organs. It can be organ specific, such as in type 1-diabetes in which pancreas is attacked, or systemic when a variety of organs and tissues are affected, such as in systemic lupus erythematosus (SLE) in which skin, muscle, heart, joints, skin, lungs, blood vessels, liver, kidneys and nervous system can be damaged [22,23,24]. Based on epidemiology studies it is estimated that between 25 and 50 million Americans have an autoimmune disease.

Deregulation of autophagy has been suggested to be implicated in autoimmune diseases. Genome-wide association studies on SLE have identified several autophagy-related susceptibility genes, including *ATG5*, *ATG7*, *PRDM1*, *DRAM1* and *IRGM* (Table 1) [25,26,27,28]. A few papers have described aberrant autophagy in B and T lymphocytes collected from peripheral blood mononuclear cells (PBMCs) from SLE patients, and from lupus mice models [29,30,31,32]. Accumulated autophagosomes and increased MaA flux have been observed in T cells from both SLE patients and Murphy Roths Large (MRL)/lymphoproliferation (lpr) or MRL/MpJ-Fas^lpr^, henceforth referred to as MRL/lpr, and the F1 hybrid of New Zeeland black (NZB) and New Zeeland white (NZW), or (NZB/W)F1 lupus mouse models [29]. These dysfunctions could be closely related to well-documented T-cell autoreactivity and abnormal TCR signaling in lupus [33]. Similarly, the increase of autophagosomes and MaA flux has been observed in B cells from PBMCs of SLE patients and NZB/W lupus mice [31]. CMA has also appears to be upregulated in MRL/lpr B splenocytes [30]. B cells are important antigen-presenting cells (APCs) in lupus. They contribute to the abnormal (auto)antigen presentation [34,35]. As summarized above, both MaA and CMA have been suggested to play an important role in antigen presentation. We have proposed that the hyperactivity of MaA and CMA, found notably in lupus B cells, contribute in a decisive manner to the aberrant (auto)antigen presentation in lupus [30,36]. It is possible that autoantigens can be substrates of both MaA and CMA. However, experimental details directly linking the irregular autophagy and altered antigen presentation in autoimmune diseases are still not available. Furthermore, one needs to take into consideration that lysosomes are dysfunctional, at least in some organs [30], which also contributes to the abnormal (auto)antigen presentation in lupus [36]. MaA in B cells has been shown to mediate autoimmunity in *Tlr7* transgenic mouse strains [37]. These findings and other data strongly suggest that the abnormalities of both autophagic pathways in immune cells are directly or indirectly linked to the autoimmune pathology of lupus.

The status of autophagy in other autoimmune diseases is less well known, likely due to the difficulty of analyzing autophagy in patient’s samples and the fact that pertinent animal models are lacking or imperfectly mimic the human disease. In this recently growing area of research, hereby we update available information summarized previously regarding autophagy in various autoimmune diseases [23]. The model systems or the type of patients’ samples tested, the methods used and the data obtained are highlighted (Table 1). Other information centered on neurological autoimmune diseases is compiled elsewhere [38].

## 2. MRL/lpr Mice as a Model for SLE

Numerous murine models have been developed to understand the cellular and genetic requirement for SLE induction, development and recurrence after asymptomatic periods. There are spontaneous lupus models, including the (NZB/W)F1 and MRL/lpr [55] mice, which display different MHC haplotypes (H-2^d/z^ and H-2^k^, respectively). Both models have been used for autophagy studies. There are also induced models, such as the pristane-induced model and the chronic graft-versus-host-disease models [56], and genetically-modified mouse models [57,58]. Several reviews have described these different models of lupus [59,60].

The MRL/lpr strain is one of the best characterized models for SLE. It develops many SLE-like features, including increased levels of autoantibodies (antinuclear, anti-double stranded (ds)DNA, and anti-Sm antibodies) and circulating immune complexes that can be pathogenic and at the origin, at least in part, of the glomerulonephritis classically visualized in these mice. The MRL background plays an important role in the development of lupus phenotype in these mice. MRL^+/+^ mice, which develop a milder and slower disease than MRL/lpr mice, while also sick, are sometimes used as mouse control for MRL/lpr mice. The recessive autosomal mutation *lpr* is responsible for the nonfunctional transcription of Fas (CD95), a member of the TNF receptor super family, leading to defects in apoptosis and an impressive amplification of the disease [59,61]. This mutation that spontaneously arose on the MRL genetic background aggravates the lupus-like symptoms in these mice, due in particular to abnormally surviving autoreactive CD4^+^ T cells and B cells in MRL/lpr. Unlike human SLE disease that mainly affects women (9:1 female to male ratio of disease incidence in human), both males and females develop SLE-like phenotype in MRL/lpr mice. In our laboratory, however, we principally work with female mice to better mimic the human (hormonal) conditions. We have privileged the MRL/lpr mouse model because with regard to (female) (NZB/W)F1 mice, lupus symptoms appear much earlier in these mice (10–14 vs. 40 weeks), and because they are much more severe than in (NZB/W)F1 mice. We should note, however, that MRL/lpr mice display some clinical and biological features that are not typically found in lupus patients or in (NZB/W)F1 mice. These are the presence of serum circulating rheumatoid factors, for example, salivary gland (SG) involvement, and peripheral cell lymphoproliferation. MRL/lpr mice also show progressive and lymphadenopathy due to the accumulation of an unusual population of CD4^−^CD8^−^CD3^+^CD45^+^/B220^+^αβ^+^ double negative (DN) T cells [62,63]. On the other hand, they develop a syndrome that in addition to be serologically similar to SLE, is pathologically close of the human disease in terms of kidney and brain involvement [64]. They also develop dermatitis and vasculitis.

We have previously reported a hyperactivity of MaA in T cells from both thymus and spleen from MRL/lpr mice [29]. Using electron microscopy, we have quantified the amount of autophagic vacuoles in splenic T cells from both MRL/lpr mice and control CBA/J mice (inbred strain from a cross of a Bagg albino female and a DBA male), and found a much higher amount of autophagosomes in MRL/lpr spleen T cells. Analyses of the MAP1LC3-II levels measured by Western blotting in the presence and absence of lysosomal inhibitors (pepstatin A and E64d) indicated that there is a higher MaA flux in MRL/lpr T cells compared to CBA/J T cells. Together, these data argue for a higher MaA activity in splenic T cells from MRL/lpr vs. control mice. In the thymus, there was no significant change regarding the amount of autophagosomes, but the MaA flux, as measured by Western blotting, was found to be increased compared to control mice [29]. Although not observed in our earlier studies, MaA activity has also been reported to be over-activated in splenic B cells of (NZB/W)F1 mice [31]. In addition to the above results related to MaA, we also identified an overexpression, in MRL/lpr mice, of CMA-specific lysosomal receptor LAMP-2A, suggesting a higher CMA activity in splenic B cells in these mice [30]. Besides the abnormality of autophagic pathways, biochemical studies showed an increase of cathepsin D protein expression, an elevated lysosomal volume and a significant raise of average lysosomal pH in the MRL/lpr B cells [30,36], strongly suggesting the existence of lysosomal dysfunction in those cells, which might be related to the aberrant autophagy activity that was observed.

As mentioned above, besides being a pertinent animal model for SLE, MRL/lpr mice can be used to mimic other autoimmune diseases. For example, the SGs of MRL/lpr mice have been used to study secondary Sjögren’s syndrome [65]. The brain of MRL/lpr mice is an excellent model to investigate elements of neuropsychiatric lupus, a severe form of SLE featured by various brain malfunctions [64,66].

## 3. Methods and Notes

In this short technical review, first we will explain how to adequately collect and purify immune cells from organs and tissues from lupus individuals (mice and patients), and then we will shortly describe the various techniques currently applied in our laboratory to measure autophagy activity in these cells (Table 2).

B and T cells display very limited cytosolic space, precluding extensive immunofluorescence studies in pooled lymphocytes and even less at the single cell level. Likely for the same inherent reasons, fluorescence microscopy approaches have seldom been used for studying autophagy in autoimmune diseases. Multispectral imaging flow cytometry (MIFC) only has been applied for studying MaA in B cells stained with anti-MAP1LC3 antibodies. MIFC is a combination of flow cytometry (FC) and fluorescence microscopy, which allows differentiating the punctate pattern of MAP1LC3-II and diffused fluorescence of MAP1LC3-I.

**General notes:**

- We highly recommend, whenever possible, to analyze specific cell subtypes rather than studying whole organ homogenates or unfractionated peripheral blood samples that contain mixed cell subsets, as the latter may exhibit very different autophagy activation status that can affect the detection of events.
- As recommended in authoritative reviews on autophagy, several different autophagic assays need to be applied to make reliable conclusions [67,68,69,70]. One single assay is by far not sufficient to determine whether the autophagy activity is abnormally increased or decreased. The number of individual samples analyzed also has to be sufficient to allow robust statistic interpretation of data.

### 3.1. Obtaining Cell Homogenates from Organs

#### 3.1.1. Obtaining Homogenates from the Spleen

Splenocytes are obtained according to standard procedures, as described for example in the series of *Current Protocols in Immunology* [71]. Briefly, spleens collected from control mice (e.g., CBA/J, C57BL/6 mice or MRL^+/+^ mice) and MRL/lpr mice are placed in a cell strainer (40 or 70 µm), mashed using the plunger end of a syringe and rinsed with Roswell Park Memorial Institute (RPMI) culture medium supplemented with 10% (*v*/*v*) fetal bovine serum (FBS) and antibiotics. Cells are centrifuged and then treated with 1–2 mL ACK [150 mM NH4Cl, 10 mM KHCO3, 0.1 mM ethylene diamine tetra acetic acid (Na2EDTA), pH 7.2–7.4] lysis buffer to obtain a single spleen cell suspension (around 10 million from CBA/J or C57BL/6 control mice and 10 to 50 million cells from MRL/lpr mice) free of red cells for further tests.

The immune cell subpopulations in the total spleen cell fraction vary in MRL/lpr mice and control mice. For example, at 12 weeks of age, the spleen cells of C57BL/6 mice consist of 60% B cells (CD3^−^B220^+^) and 30% T cells (CD3^+^B220^−^), whereas the MRL/lpr splenocyte fraction is composed of 30% B cells (CD3^−^B220^+^), 20% T cells (CD3^+^B220^−^), and 10–40% DN T cells.

B and T cells can then be isolated from the total splenocytes using standard B and T cell isolation kits that are commercially available. We have chosen kits from Miltenyi Biotec (Bergisch Gladbach, Germany) (Pan B Cell Isolation Kit II, mouse, 130-104-443; Pan T Cell Isolation Kit II, mouse, 130-092-130), as they allowed better enrichment and higher purity of sub-populations of interest in our experiences. The purity of B cells or T cells isolated from MRL/lpr mice is around 90%, while that of B or T cells purified from CBA/J or C57BL/6 mice, is usually higher than 95%.

**Notes:**

**Figure 2:**
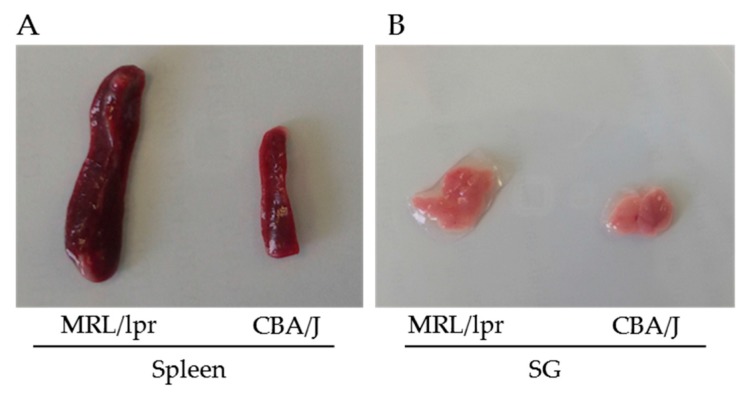
Images of spleens (**A**) and SGs (**B**) from MRL/lpr lupus mice and CBA/J control mice. The MRL/lpr and CBA/J mice were 26 and 28 weeks old, respectively.

- Some fat tissues are commonly found in the splenic cell suspension prepared from MRL/lpr mice (rarely observed in the case of control CBA/J or C57BL/6 mice). They can be removed by filtering cell fraction through a 40-µm cell strainer, according to our experiences.
- The spleen of MRL/lpr mice is usually 4–6 times the size of that of CBA/J mice at the same age (Figure 2A). It is important to take into consideration this huge difference, as for certain experiments pooling 2–3 spleens from CBA/J or C57BL/6 control mice will be required to have enough control splenocytes.
- The final purity of B or T cells should always be checked as it has been sometimes observed, for example, that depending on the isolation kits, 5–10% DN T cells can remain in the MRL/lpr B cell fraction in our experiences.

#### 3.1.2. Obtaining Homogenates from Lymph Nodes

The method developed to obtain cell suspension from lymph nodes (LNs) is identical to the one used above to prepare splenocytes from spleens [72]. Enzyme digestion is not required in order to obtain the immune cells from LNs. The LNs that are easier to collect are the mesenteric, brachial and inguinal nodes. The other LNs are generally much smaller. In order to obtain as many cells as possible, we routinely obtain all LNs to prepare the cell suspension. The amount of cells that can be obtained from all LNs is comparable to that obtained from spleens. However, depending on the purpose of study, one particular type of LNs can be isolated. Similar to spleen, in comparison to control mice, there is a dramatic increase of LN size in MRL/lpr mice. The number of B and T cells in LNs varies importantly among studies. This notably relies to the degree of immune cell activation. In a past study [73], we studied the activated state in LNs of CD4^+^ T cells from MRL/lpr and CBA/J control mice. This was tested by analyzing the expression of CD44 and CD62L molecules at the surface of LN CD4^+^ cells by flow cytometry. In unprimed seven-week-old MRL/lpr mice, the frequency of activated CD4^+^ T cells (CD44^hi^/CD62L^hi^) and memory CD4^+^ T cells (CD44^hi^/CD62L^lo^) was significantly increased compared to the corresponding cell subsets in CBA/J mice (13% vs. 3%, and 20% vs. 5%, respectively).

The isolation of B and T cells from LNs can be done using commercial T cell isolation kit (such as Pan T Cell Isolation Kit II, mouse, 130-092-130, Miltenyi Biotec,). Several reviews have been published which describe the conditions for isolating dendritic cells [70,74] and stroma cells [75].

#### 3.1.3. Obtaining Homogenates from Salivary Glands

Isolation of SGs should be performed with a fine touch for avoiding their alteration and also collecting unwanted tissues. There are three major paired SGs in the mouse (as also in many other species), which are submaxillary (submandibular), parotid, and sublingual SGs, and minor SGs [76]. The minor SGs are located in the oral submucosa and tongue, and are missed, in general. The three other major SGs are closely associated and interconnected. They are located in the subcutaneous tissue of the ventral neck area. The lobulated submaxillary (submandibular) are the largest SGs. Submaxillary SGs of healthy male mice are larger and more opaque than those of females. A single excretory duct from the anterior dorsal surface of each gland opens on the floor of the oral cavity posterior to the incisor teeth.

Once collected, each SG is immediately digested by 2 mL RPMI medium supplemented with 2% (*v*/*v*) FBS, collagenase D (1 mg/mL; Roche, Basel, Switzerland, 11088866001) and DNase I (50 µg/mL; Roche, 10104159001) at 37 °C under gentle agitation. The tissues that remain after 1 h of treatment are mashed using the plunger end of a syringe on a cell strainer (70 µm). Two milliliters ACK buffer is then added to the resulting cell suspension of each gland to lyse red blood cells. Eight Milliliters RPMI medium supplemented with 10% (*v*/*v*) FBS is then added to stop the lysing of ACK and cells are centrifuged at 320× *g* for 5 min. Around 0.13 million cells can be obtained from 1 mg of SG. 

In CBA/J SGs, there are no immune cells, whereas in MRL/lpr SGs, there is a characteristic infiltration of immune cells, which is composed of about 4% CD4^+^ T cells, less than 4% of CD8^+^ T cells and less than 0.1% of CD19^+^ B cells (Li and Muller, unpublished data).

**Notes:**

- The size of SGs from MRL/lpr mice is usually 1.5–2 times larger compared to that of SGs from CBA/J or C57BL/6 mice at the same age, depending on the severity of the disease (Figure 2B).
- The concentration of collagenase D and DNase needs to be optimized, as an excessive enzyme concentration can induce a loss of cell viability and a too low enzyme dose will obviously lead to insufficient digestion.
- When SG cell suspensions are prepared for FC measurement, it is essential to add EDTA in the FC buffer (such as PBS supplemented with 2% *v*/*v* FBS) to avoid cell aggregation and ensure single cell suspension. We have determined that 0.3 mM EDTA is the optimal concentration as higher concentration causes toxicity and a lower concentration is insufficient to separate cells.

#### 3.1.4. Isolating Peripheral Blood Mononuclear Cells from Patient’s Blood

PBMCs can be obtained from human blood by standard Ficoll or Ficoll–Paque methods using density gradient centrifugation [77]. Briefly, blood is first diluted 1:1 in PBS; then, at room temperature, 30 mL diluted blood is carefully layered over 10 mL of Ficoll, e.g., Histopaque^®^-1077 (Sigma-Aldrich, St. Louis, MO, USA, 10771) and tubes are centrifuged at 1300× *g* for 20 min at room temperature in a swining-bucket rotor (Eppendorf, Hamburg, Germany, 5810R) without brake. The opaque interface that contains PBMCs is then carefully collected using a Pasteur pipette. The last step consists to wash PBMCs with PBS or RPMI medium to eliminate remaining Ficoll reagent.

The percentage of different sub-populations in the PBMC fraction varies somewhat among blood donors. In general, the PBMC fraction is composed of 40–50% CD4^+^ T cells, 20–30% CD8^+^T cells and 5–15% B cells. It is possible to isolate these populations using kits, such as B cell isolation kit II, human (Miltenyi Biotec, 130-091-151) and Pan T cell isolation kit II, human (Miltenyi Biotec, 130-091-156). The purity of cells obtained using these kits, is greater than 90% as determined by FC.

**Notes:**

- In the case of blood samples taken from lupus patients, we have occasionally observed a large amount of red blood cells remaining in the PBMC layer after centrifugation in Ficoll. In this case, an additional step of ACK lysis has been included after the Ficoll step for lysing these red blood cells.
- In the case of CD4^+^ T cell isolation from blood, instead of performing a Ficoll density gradient centrifugation, RosetteSep^TM^ Human CD4^+^ T Cell Enrichment Cocktail (Stemcell Technologies, Vancouver, Canada, 15062) can be added directly to the whole blood to isolate CD4^+^ T cells by negative selection.
- Note that the number of B cells recovered from the blood of lupus patients is usually very low (2–5 × 10^6^ cells from around 40 mL of blood). This is probably related to the immunosuppressive treatments given to patients with lupus. This considerably reduces the number of assays that can be performed. Therefore, it is important to miniaturize the assays to maximum (without losing too much sensitivity and specificity) and prioritize the tests that will be carried out for the autophagy analysis.

### 3.2. Measurement of Macroautophagy by Electron Microscopy

**Figure 3:**
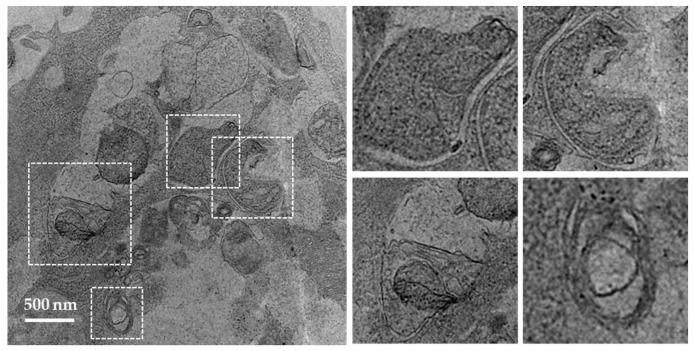
Transmission electron microscopic images of autophagosomes in SG cells. SG cells were isolated from MRL/lpr mice and treated with pepstatin A and E64d to block the degradation of autophagosomes. Autophagosomes with double membrane structures can be identified in the white dashed squares in image on the left panel, and the zoomed images of individual autophagosomes are presented on the right panel.

It is widely accepted that electron microcopy represents one of the most accurate method for detecting autophagy and quantifying accumulation of autophagic vesicles [70]. The general procedure consists to fix cells with glutaraldehyde and post-fix them in osmium tetroxide, followed by ethanol dehydration, embedding in the resin, and cutting of ultrathin sections. For the detailed experimental conditions, see [78]. Vacuoles (usually 0.5–1 µm in diameter) can be identified as autophagosomes when meeting at least two of the following criteria: double membrane, absence of ribosomes at the cytosolic sides of the vacuole, similar luminal density as that of cytosol, visible organelles or parts of organelles in their lumen [29,79]. Vacuoles of similar size but with single membrane containing dense or clear amorphous material can be considered as autolysosomes. Autophagosomes present in the SG cells collected from MRL/lpr mice are shown in Figure 3 as an example (Li and Muller, unpublished). The occurrence of “autophagic vacuoles” or “autophagic compartments” can be quantified as previously described [29].

**Notes:**

- For statistical reasons, a minimum of 50 cell sections per condition should be examined.
- It is recommended to examine grids prepared from different resin blocks to avoid counting the same cells several times.

### 3.3. Measurement of Macroautophagy by Western Blot

**Figure 4:**
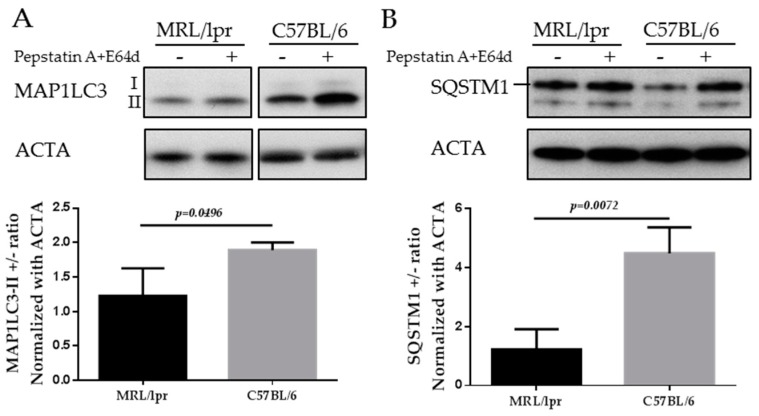
Western blots of MAP1LC3 and SQSTM1 in the SGs of MRL/lpr mice and C57BL/6 control mice. SG cells were isolated from MRL/lpr and C57BL/6 female mice, followed by starvation for 12 h in the presence or absence of lysosomal inhibitors (pepstatin A+E64d). Cells were then subjected to SDS-PAGE and Western blot. Two autophagy markers are shown in the figure: MAP1LC3 (**A**); and SQSTM1 (**B**). Actin-α (ACTA) was used as a loading control. Abbreviations: SDS-PAGE, Sodium dodecyl sulfate polyacrylamide gel electrophoresis.

The mammalian protein MAP1LC3 is a widely accepted marker for characterizing autophagosomes. The detailed usage, caution and pitfalls of MAP1LC3 as a MaA marker have been extensively reviewed elsewhere [70,80,81]. MAP1LC3 presents in two forms, namely MAP1LC3-I, which is cytoplasmic, and MAP1LC3-II, which is associated to the membrane of autophagosomes. Therefore the amount of MAP1LC3-II correlates to the number of autophagosomes, while only the turnover of MAP1LC3-II corresponds to the activity of MaA, also called “MaA flux”. The turnover of MAP1LC3-II can be assessed by comparing the difference of the MAP1LC3-II in the presence and absence of lysosomal inhibitors (pepstatin A and E64d, chloroquine and others). In the absence of lysosomal inhibitors, the MAP1LC3-II on autophagosomes will be quickly degraded once inside lysosomes. By adding lysosomal inhibitors, the degradation of MAP1LC3-II is inhibited and therefore the amount of MAP1LC3-II delivered to lysosomes via autophagy can be monitored. As illustrated below in the images of MAP1LC3 blots, the MAP1LC3-II level is increased in SG cells of C57BL/6 mice incubated with lysosomal inhibitors (Figure 4A). In contrast, in the SGs of MRL/lpr mice, there is no accumulation of MAP1LC3-II in the presence of lysosomal inhibitors, indicating defective autophagy in the SGs of MRL/lpr mice (Li and Muller, unpublished).

SQSTM1/p62 is selectively incorporated into autophagosomes through binding to MAP1LC3 and is degraded through MaA. Therefore, the cellular expression level of SQSTM1 negatively correlates with MaA activity. As shown in Figure 4B, the level of SQSTM1 is lower in C57BL/6 SGs cells compared with MRL/lpr SGs (Li and Muller, unpublished). This result reinforces the MAP1LC3 data since an accumulation of SQSTM1 is suggestive of a defective MaA.

In practice, specific procedures have been detailed in numerous articles and technical reviews [82,83]. The protocols routinely used in our own studies to visualize and quantify MAP1LC3-II conversion and SQSTM1 accumulation have been described elsewhere [29]. The antibodies we selected are listed in Table 3.

**Notes:**

- Serum deprivation is widely used as a stimulus to induce autophagy. It is important to keep in mind that B cells are much more sensitive to serum withdrawal compared to T cells and many other cells. Therefore, we do not recommend incubating B cells in serum free media for more than 4 h, while more than 12 h serum withdrawal can be used for T cells and SG cells.
- The limitation of using Western blotting for B cells isolated from the blood of lupus patients relies to the number of recovered cells, which is generally too low to perform several conditions (including the controls run in the presence or absence of lysosomal inhibitors). In our experience, it is optimal to use one million cells (or around 20 µg protein) per condition/lane to obtain good signals, while commonly only 2–5 million B cells are recovered from lupus patients. It is therefore more feasible to use alternative approaches such as FC-based methods, as in the latter, much fewer cells are needed per condition.
- Loading controls are essential for proper interpretation of Western blots. They are important to assess the total proteins that have been loaded in each lane across the gel, thus allowing a more accurate blot calibration. Loading controls also permit gel conditions to be checked and compared from gel to gel.
- Regarding the type of proteins used as loading controls, we noticed that actin-α, but not actin-β, can be readily used as a loading control of SG extracts, whereas actin-β, but not actin-α, can be used when splenocytes are studied. Among usual loading controls, attention should be paid not to use a marker that is affected by autophagy alteration; for example, in certain cases, glial fibrillary acidic protein (GFAP) is not an appropriate control.

### 3.4. Measurement of Macroautophagy by Flow Cytometry

#### 3.4.1. Measurement of MAP1LC3 by Flow Cytometry

Traditional FC can only measure the total cellular fluorescence of MAP1LC3 antibody staining, leaving MAP1LC3-I and MAP1LC3-II undistinguished. This can be overcome by using a MIFC, such as Amnis ImageStream^X^ instrument (EMD Millipore, Seattle, MA, USA), which can discriminate the punctate MAP1LC3-II from the diffused cytosolic MAP1LC3-I [31,84]. The number of MAP1LC3^+^ punctates per cell is calculated to represent the number of autophagosomes. However, there are limitations for this method. Firstly, not every laboratory is equipped with this instrument. Secondly, five million cells are required for each sample, and this number could be too high for conditions where cell number is limited.

We have obtained rather good results using FlowCellect^TM^ Autophagy MAP1LC3 antibody-based assay kit (Millipore, Billerica, MA, USA) following the manufacturer’s instructions (see below). This method overcomes the problem that classical FC measurement cannot distinguish MAP1LC3-I from MAP1LC3-II, by adding a permeabilization solution that extracts the soluble cytosolic MAP1LC3-I while protecting the MAP1LC3-II form, which is sequestered in the autophagosomes. In this method, unsorted PBMCs collected from blood can be used, as surface staining required to distinguish certain cell populations can be included to measure the number of autophagosomes in multiple cell populations. We have calibrated this assay using total splenocytes from CBA/J and MRL/lpr mice. Only 0.1 million cells were used per condition, and we included samples with and without lysosomal inhibitors (pepstatin A and E64d). This method has been used to measure the activity of MAP1LC3-II level in splenic macrophages in MRL/lpr as well [42].

CYTO-ID Autophagy detection kit (Enzo Life Sciences, Lausen, Switzerland) is another commercially available tool to measure autophagy by FC. The probe used in this kit is a cationic amphiphilic tracer dye that primarily stains autophagosomes, while staining lysosomes minimally. This dye has been successfully used in both cell lines and primary cells from human and mouse origin (summarized in the company’s website). It can be used in conjunction with surface markers that discriminate different cell populations [85,86].

We deliberately do not describe here other modes of flow cytometric measurement, such as those exploiting fluorescence probes GFP-MAP1LC3 [67,87] or mCherry-GFP-MAP1LC3 [88]. These methods require transfection or transduction to introduce the fluorescence proteins, which is usually inapplicable in our autoimmunity projects that mostly examine primary cells isolated from organs and rarely cell lines that are easily tranfectable.

**Notes:**

**Figure 5:**
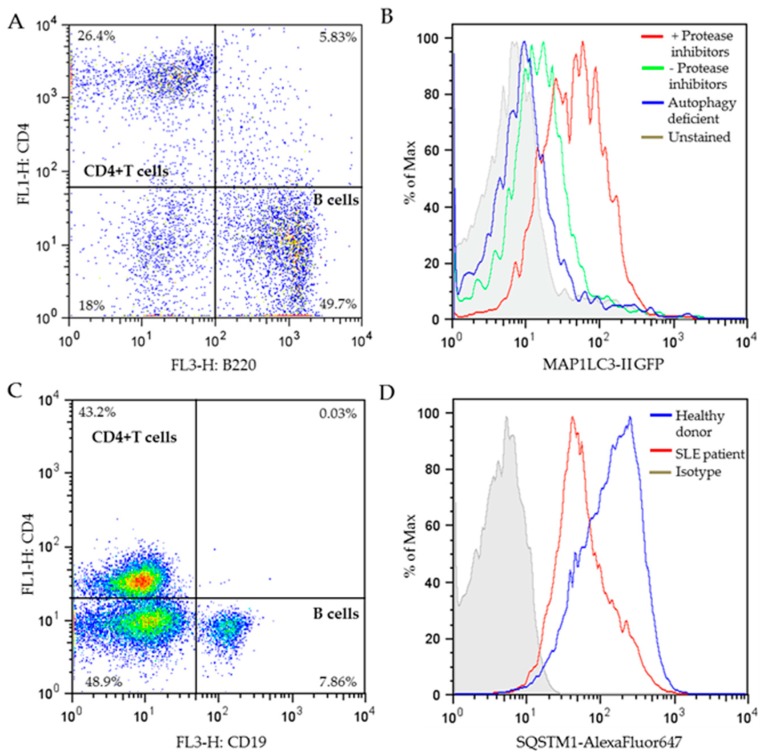
Flow cytometry measurement of autophagy markers. (**A**) Dot plot of MRL/lpr spleen cells after surface staining of CD4-FITC/B220-APC. The percentages of individual cell populations are indicated. (**B**) Representative histograms of MAP1LC3-II GFP fluorescence in stimulated CD4^+^ T cells from MRL/lpr spleen cells (gated from the dot plot of A) in the presence or absence of protease inhibitors (pepstatin A+E64d), or stimulated CD4^+^ T cells from autophagy-deficient mice (Atg5f/f dLck-cre mice; unpublished). Cells were stained using the FlowCellect^TM^ Autophagy MAP1LC3 antibody-based assay kit according to manufacturer’s instructions, or left unstained as indicated. (**C**) Dot plot of PBMCs from SLE patients after surface staining of CD4-FITC/CD19-APC. The percentages of separate cell populations are indicated. (**D**) Representative histograms of SQSTM1-AlexaFluor^647^ fluorescence in stimulated CD4^+^ T cells (gated from the dot plot of C) from the PBMCs of a healthy donor and a patient with SLE stained with SQSTM1 antibody or control isotype. The selected patient displayed a relatively high SLEDAI severity score of 20 on a scale of 0–105. CD4^+^ T cells were stimulated with anti-CD3 and anti-CD28 antibodies. APC, allophycocyanin; FITC, fluorescein isothiocyanate; GFP, green fluorescent protein; PBMCs, peripheral blood mononuclear cells; SLEDAI, systemic lupus erythematosus disease activity index.

- The first advantage of measuring autophagosomes by MAP1LC3 staining with FC is that a limited number of cells is needed (around 0.1 million per condition, except in the imaging cytometer), compared with the number of cells required for Western blot (0.5–1 million cells per condition).
- Secondly, surface staining to distinguish cell populations (Figure 5A) can be done before permeabilization and immunostaining of MAP1LC3; therefore, no prior cell isolation is needed. We highly recommend FC measurement of MAP1LC3 staining in combination of surface cell markers when the number of cells that are available is low, such as it is the case when B cells from lupus patients are analyzed.
- It is very important to avoid high background staining, which we have observed when some MAP1LC3 staining kits are used. We strongly advise the users to include the following controls when calibrating the MAP1LC3 assay:
  − positive controls: cells treated with lysosomal inhibitors that block degradation of autophagosomes and therefore increase the amount of MAP1LC3-II;
  negative controls: cells in which the initiation of autophagy has been blocked, e.g., cells from autophagy deficient mice, and unstained cells.

The appropriate autophagy-detecting reagent is the one that allows separation of these controls. As illustrated in Figure 5B, the level of MAP1LC3-II (stained using the FlowSellect^TM^ kit) is much higher in CD4^+^ T cells incubated in the presence of lysosomal protease inhibitors, compared to that in the absence of lysosomal protease inhibitors, while the MAP1LC3-II level in the CD4^+^ T cells from autophagy-deficient mice is as low as cells that were left unstained, indicating the staining of FlowSellect^TM^ kit is successful.

#### 3.4.2. Measurement of SQSTM1 with Flow Cytometry

Various specific antibodies have been developed for measuring SQSTM1 level by FC. In line with their observation that the number of autophagosomes was increased in (NZB/W)F1 B cells compared to normal B cells, Clarke et al. observed a decrease of SQSTM1 expression in B cells of lupus NZB/W mice in comparison to B cells collected from control C57BL/6 mice, using FC measurement of SQSTM1 antibody staining [31].

In our laboratory, we have successfully carried out FC measurement with SQSTM1 antibody in stimulated PBMCs from healthy donors and lupus patients. PBMCs were first stained with surface markers for B cells and T cells to differentiate these cell subtypes (Figure 5C), followed by permeabilization and staining of SQSTM1 antibody. CD4^+^ T cells were gated from the dot plot of Figure 5C, and the SQSTM1 expression level of CD4^+^ T cells was analyzed and illustrated in Figure 5D. The results show much lower SQSTM1 expression in SLE patient’s CD4^+^ T cells compared to healthy donors, supporting that in average, T cells from lupus patients display higher MaA activity. This result is highly consistent with the CD4^+^ T cells “hyperautophagic” phenotype reported previously in both lupus prone mice and patients [29].

**Notes:**

- Autophagic flux can also be evaluated by measuring the difference of SQSTM1 level in the presence and absence of lysosomal inhibitors [31].

### 3.5. Measurement of CMA by Western Blot

There are relatively few relevant assays currently designed to readily study and analyze CMA and, nowadays, no commercial quantitative assay is available. An important contribution was made by Cuervo and colleagues who developed several sophisticated functional assays and steady-state measurement to evaluate CMA activity [89,90,91].

A first functional assay set up to follow CMA is based on a “pulse and chase” experiment in which cells are pulsed with a radiolabeled amino acid residue to preferentially label long-lived proteins in the presence of lysosomal inhibitors or inhibitors for MaA. The release of free radiolabeled amino acids in the culture media during the chase time can be measured as the rate of proteolysis. The difference of proteolysis measured between untreated cells and cells treated with lysosomal inhibitors corresponds to the total lysosomal degradation; the difference of proteolysis monitored between untreated cells and cells treated with MaA inhibitors represents the lysosomal degradation through MaA; the difference of the total lysosomal degradation and the degradation through MaA is the degradation through CMA (and also microautophagy) [89]. A second functional assay is based on photoconvertible CMA reporters, which are fluorescence proteins with an addition of the KFERQ motif, allowing its degradation through CMA. The advantage of using photoconvertible probes is that they could distinguish a subset of reporter converted to a different fluorescence from the constantly newly synthesized reporters (original fluorescence). Upon CMA activation, the former forms lysosomal puncta while the latter shows diffused cytosolic pattern [92]. Using this method, we have demonstrated that a therapeutic phosphopeptide currently evaluated to treat lupus patients (P140/Lupuzor^TM^) exhibits inhibition effect to CMA [30,36]. This method has been better established in cell lines [30,92], however transfections of plasmid carrying the CMA reporter in primary cells are often challenging. The third and ultimate functional assay that was described consists to reconstitute CMA in vitro using isolated lysosomes (see methods of isolation of lysosomes adapted for CMA measurement in [89,91,93]). The differences of CMA substrates, such as GAPDH, recovered after incubation with lysosomes in the presence or absence of lysosomal protease inhibitors or protease K (that digests the substrate at lysosomal surface) could represent the binding, association and uptake steps of CMA [89]. Technical details have been described in [89,90,91]. This method requires a large amount of cells from tissue/organ samples, which could be difficult to obtain in certain settings, in particular when examining blood samples or purified cell subsets.

The steady-state CMA measurement includes examining the amount of key CMA components, which is however an indirect way to measure CMA and should be complemented with functional assays as those described above. The lysosomal amount of LAMP-2A and HSPA8 usually correlates with CMA activity and can be measured with Western blot as indirect indicators of CMA activity. The perinuclear localization of HSPA8-positive lysosomes can also be used as an indirect indication of CMA activation. In the context of autoimmune diseases, measuring the amount of LAMP-2A has been used to indirectly measure CMA activity in lupus-prone and healthy mice [30]. An unexpected overexpression of LAMP-2A was repeatedly detected in spleen lupus B cells as the disease progresses, indicating hyperactivity of CMA in these mice [30].

**Notes:**

**Figure 6:**
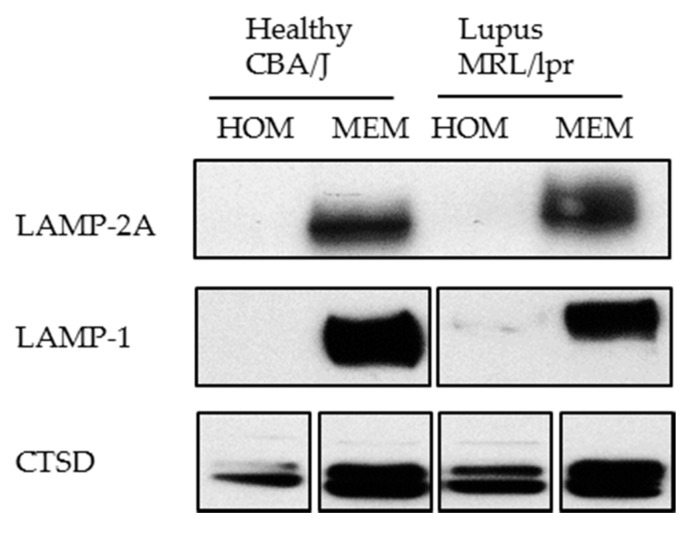
Western blotting of LAMP-2A in the homogenate (HOM) and membrane (MEM) fractions prepared from CBA/J and MRL/lpr spleen cells. Twenty microgram of protein was loaded per lane. The result shows a good detection of LAMP-2A in the MEM fraction in which lysosomes are enriched, but no visible signal in HOM fraction. LAMP-1 (lysosomal membrane associated protein type 1) and CTSD (cathepsin D) are lysosomal markers showing enrichment of lysosomes in the MEM fractions.

- It should be kept in mind that three variants of LAMP-2 exist and share the same lysosomal lumen region [90]. Therefore, for detecting LAMP-2A only, it is important to use antibodies that specifically detect the cytosolic tail of LAMP-2A. Specific antibodies can be raised as the 12 amino acid residues on the cytosolic side of LAMP-2A significantly differ from those encompassed in its variants LAMP-2B and C.
- We have experienced that sometimes LAMP-2A cannot be detected using whole cell lysate (homogenate). In this case, for studying LAMP-2A, we recommend preparing a membranous cell fraction with enriched lysosomes and mitochondria through classical step-wise centrifugation. This membrane cell fraction can be prepared by following the protocol published in Kaushik and Cuervo, 2008 (Section 3.1, Step 1–4) [91], however without going through the long and challenging process of preparing the highly purified lysosomes. Figure 6 illustrates our own data: LAMP-2A level is not detectable in cell homogenates (HOM), while there is a strong signal in the membrane fraction (MEM).

## 4. Conclusions

This review aims at providing some technical information for studying autophagy (both MaA and CMA) in lupus setting using MRL/lpr mice as a model. We also describe some methods we use in routine with human cells. As it is detailed above and summarized in Table 4, all the techniques have their pros and cons. Therefore, it is essential to combine several techniques to make robust conclusions. We and others have already obtained important information on the activity of MaA in both B and T cells in autoimmune mouse models and patients. The next step will be to identify the precise links between aberrant MaA and autoimmune responses both in vitro and in vivo. In contrast to what is known in MaA, data made available on the CMA activities in various immune cell types are still scarce. Pertinent probes that could detect directly CMA activity in vivo and specific inhibitors or activators of CMA are still lacking [10]. For both MaA and CMA, new mouse models that carry features of SLE and with deletion of genes involved in these pathways would be useful in order to better understand the role of these two major autophagic pathways in SLE and hopefully also in other autoinflammatory diseases.

We hope that this review and the many technical details we list herein will be useful more widely, to researchers who would like to assess the status of autophagy in chronic and acute inflammatory diseases, both in animal models and patient’s samples. All kinds of information on the implication of autophagy in this context would be of great importance for the development of novel, specific and safe therapies that target autophagy [10,23,36].

## Figures and Tables

**Table 1:** List of autoimmune diseases with autophagy abnormalities and of the type of animal model organs/tissues or patient’s samples tested.

Autoimmune Diseases	Autophagy Abnormalities	Methods	Model Systems or Patient Samples Tested	Ref.
**Systemic lupus erythematosus**	Associated genes: *ATG5*, *ATG7*, *PRDM1*, *DRAM1*, *IRGM*	N/A	N/A	[25,26,27,28]
	Accumulation of autophagosomes and increased MaA flux in T splenocytes	WB, EM	MRL/lpr and (NZB/W)F1 mice (thymus, spleen)	[29]
	Increased amount of autophagosomes in T cells	EM	Patients (blood)	
	Increased MAP1LC3 puncta, decreased SQSTM1/p62, and increased MaA flux in B cells	MIFC, FC	NZB/W F1 mice (spleen, bone marrow)	[31]
	Increased MAP1LC3 puncta and increased MaA flux in B cells	MIFC	Patients (blood)	
	Increased mRNA of Beclin-1, MAP1LC3 and SQSTM1 in PBMCs	qPCR	Patients (blood)	[39]
	Increased expression of *ATG5*, *ATG12* and *BECN1* in macrophages	pPCR	Induced lupus mice (spleen, kidneys) and patients (blood)	[40]
	Increased HSPA8 expression in B cells	WB, FC, qPCR	MRL/lpr mice (spleen)	[41]
	Increased LAMP-2A and CTSD expression in B cells; defective lysosomes in B cells	WB, FC	MRL/lpr mice (spleen)	[21]
	Increased MAP1LC3-II protein level	FC	MRL/lpr mice (spleen)	[42]
**Secondary Sjögren’s syndrome**	Defective autophagy in salivary glands	WB, EM	MRL/lpr mice (salivary glands)	Li & Muller, unpublished
**Crohn’s disease**	Associated genes: *ATG16L1*, *IRGM*	N/A	N/A	[43,44,45,46]
**Rheumatoid arthritis**	Associated genes: *ATG5*, *ATG7*, *BECN1*	N/A	N/A	[47,48]
	Increased protein expression of ATG7 and BECN1	WB, IHC	Patients (bones)	[47]
	Increased BECN1, ATG5, MAP1LC3 mRNA expression; increased MAP1LC3-II protein level	qPCR, IH, WB	Patients (synovial tissues)	[49]
	Decreased SQSTM1 protein expression	WB	Patients (synovial tissues)	[50]
**Polymyositis**	Increased levels of MAP1LC3-II and decreased level of p70S6 kinase	WB	Patients (muscle)	[51]
**Multiple sclerosis**	Associated gene: *ATG5*	N/A	N/A	[52]
	Increased mRNA and protein level of ATG5	qPCR, WB	EAE mice (blood) and patient (blood and brain)	[52]
	Decreased expression of *ATG16L2* and *ATG9A* genes and increased expression of *ULK1* gene	qPCR	Patient (blood)	[53]
**Type 1 diabetes**	Decreased MAP1LC3 and ATG5/12 protein level	WB	Induced diabetic mice (heart)	[54]

ATG, autophagy related; BECN1, beclin-1; CTSD, cathepsin D; EAE, experimental autoimmune encephalomyelitis; EM, electron microscopy; FC, flow cytometry; IHC, immunohistochemistry; MAP1LC3/LC3, microtubule associated protein 1 light chain 3; MaA, macroautophagy; MIFC, multispectral imaging flow cytometry; N/A: not applicable; qPCR: quantitative polymerase chain reaction; SQSTM1/p62, sequestosome-1; ULK1, Unc-51 like-autophagy activating kinase 1; WB, Western blot.

**Table 2:** Techniques currently used in routine in our laboratory to evaluate MaA activity in different organs and tissues from autoimmune mice and patients.

	Mice			Human
	Spleen	Lymph Node	Salivary Gland	Blood
EM	Yes	No	Yes	Yes
WB	Yes	Yes	Yes	Yes
FC	Yes	No	No	Yes

EM, electron microscopy; FC, flow cytometry; WB, Western blot.

**Table 3:** References of antibodies used in our settings to analyze autophagy activity by WB and FC in various organs or blood from mice and patients with lupus.

Antibodies		Company, References	Organs or Tissues Tested
**WB**	MAP1LC3	MBL International Corporation, M186-3	Mice (spleen, LN, SG), human (blood)
	SQSTM1	Abcam, ab109012	Mice (spleen, SG), human (blood)
	ATG5/ATG12	Abcam, ab155589	Mice (spleen, SG), human (blood)
	LAMP-2A	Abcam, ab18528, polyclonal; Abcam, ab125068 monoclonal	Mice (spleen, LN, SG), human (blood)
	HSPA8	Abcam, ab51052	Mice (spleen, SG), human (blood)
	HSP90	ENZO, ADI-SPA-831	Mice (spleen, SG), human (blood)
	Actin-β HRP	Abcam, ab49900	Mice (spleen), human (blood)
	Actin-α HRP	Abcam, ab203696	Mice (SG)
**FC**	MAP1LC3-FITC (FlowCellect^TM^)	Millipore, FCCH10071	Mice (spleen), human (blood)
	SQSTM1 AlexaFluor 647	MBL International Corporation, M162-A64	Mice (spleen), human (blood)
	HSPA8-PE	Abcam, ab65170	Mice (spleen), human (blood)
	HSP90-PE	Abcam, ab65171	Mice (spleen), human (blood)

FC, Flow cytometry; FITC, fluorescein isothiocyanate; PE, phycoerythrin; WB, Western blotting. Informations of companies: Abcam, Cambridge, United Kingdom; ENZO life sciences, Lausen, Switzerland; MBL International Corporation, Woburn, MA, United States; Millipore, Billerica, MA, United States.

**Table 4:** The pros and cons of the techniques described in this review.

	Pros	Cons
EM	- It is the best method to visualize the double membrane structure of autophagosomes.	- Quantification of autophagic vesicles through EM is time/sample-consuming. - It is prone to be subjective.
WB	- It is the best way to distinguish the two forms of MAP1LC3 and semi-quantify the MaA flux.	- Prior cell isolation is required in order to study cell subsets in the organs or blood. - 0.5–1 million cells per condition are needed.
FC	- Immunostaining of surface markers can be carried out at the same time, in order to examine autophagy on cell subsets. - A small number of cells (0.1 million cells) is needed. - Time saving.	- Some kits are not able to distinguish the two forms of MAP1LC3.
MIFC	- It can distinguish the punctate MAP1LC3-II from the diffused MAP1LC3-I. - It is both quantitative and qualitative.	- A large number of cells (5 million cells per condition) are needed.

EM, electron microscopy; FC, flow cytometry; MIFC, multispectral flow cytometry; WB, Western blot.
